# Editorial: Pathological Changes in Erythrocytes During Inflammation and Infection

**DOI:** 10.3389/fphys.2022.943114

**Published:** 2022-06-17

**Authors:** Janette Bester, Albe Carina Swanepoel, Ursula Windberger

**Affiliations:** ^1^ Department of Physiology, University of Pretoria, Pretoria, South Africa; ^2^ Centre of Excellence for Nutrition, North-West University, Potchefstroom, South Africa; ^3^ Core Facility Laboratory Animal Breeding and Husbandry, Medical University Vienna, Vienna, Austria

**Keywords:** erythrocyte (RBC), inflammation, COVID-19, malaria, rheology

This Research Topic focused on the pathological changes in erythrocytes during infection or inflammation. It has been shown that during inflammation, erythrocytes can undergo both biochemical, as well as biophysical alterations (Pretorius, 2018). Biochemical changes can be seen as disruptions in the molecular arrangement of the plasma membrane and erythrocyte function, whereas biophysical changes are noted as changes to the general structural arrangement and erythrocyte morphology, translating to changes in erythrocyte mechanics (Bester and Pretorius, 2016; Pretorius et al., 2016; Page et al., 2018). Generally, inflammatory molecules interact with erythrocyte membranes, leading to structural changes (Lang et al., 2006; Lang et al., 2012).

The articles published in this Research Topic elucidated how inflammation and infection play a role in altering erythrocyte metabolism, mechanics, and function. One of these articles is by Maruyama et al., which reviewed the rheological abnormalities of erythrocytes subjected to oxidative inflammation. It was noted that in diabetes mellitus the erythrocytes show an enhanced prothrombotic tendency, based on changes in cell deformability, aggregability, and membrane fluidity. Also, in hypertension, the reduction of erythrocyte deformability becomes proportional to the mean blood pressure of patients on treatment. In conclusion, the authors stated that the impairment of erythrocytes in circulation disrupts systemic microcirculation and induces tissue hypoxia.

Another two articles addressed the changes of erythrocytes seen in COVID-19 infected patients and associated disease severity (Bouchla et al.; Piagnerelli et al.). Bouchla et al. indicated that the virus-induced erythrocyte oxidative stress increased intracellular Ca^2+^ levels which resulted in higher erythrocyte fragility. In the study from Piagnerelli et al., it was shown that there was no change in erythrocyte deformability in relation to disease severity, however, these cells did show alterations in shape and were spherically shaped on day-7 of infection. The authors connected the preservation of normal deformability with the microcirculatory response to hypoxia in their patients. In essence, these two articles indicate that notable changes can occur in erythrocytes in patients infected with COVID-19 which contribute to the complications associated with the disease.

On the other hand, multiple erythrocyte alterations have been described during malaria, including decreased deformability that correlated with disease severity (Malaria, 2005; Jauréguiberry et al., 2014). Anemia has been shown to be the leading cause of morbidity and mortality in malaria, some of them due to treatment (Price et al., 2001). Here Chambrion et al. explored the changes in infected erythrocyte subpopulations of patients under treatment with artemisinin derivatives (AD). The data showed that infection by *P. falciparum* induced modification of the proteins and the lipids located in the erythrocyte plasma membrane and the cytosol, subsequently causing reduced deformability related to splenic retention and clearance. These modifications were still prevalent after treatment. Not only did infection and treatment profoundly affect certain erythrocyte subpopulations it also had a large effect on never infected erythrocyte populations. Chambrion et al. explained that this might be why the magnitude of never infected erythrocytes lost is frequently higher than infected erythrocytes in malaria anaemia. In conclusion, it was said that infection with *P. falciparum,* artesunate treatment, and pitting triggered alterations in the biochemical properties of circulating erythrocytes, and that early post-treatment anaemia is mostly due to the clearance of never infected erythrocytes.

From this Research Topic, it is clear that erythrocytes are affected in many conditions and contribute to the complications associated with these conditions. It is obvious that erythrocyte changes are triggered by endogenous challenges induced by the different conditions and that it contributes to the related complications. Nonetheless, our knowledge about mechanisms underlying these changes and the implications are still incomplete (Lang et al., 2012).
